# Artificial Intelligence Applied to Chest X-Ray Images for the Automatic Detection of COVID-19. A Thoughtful Evaluation Approach

**DOI:** 10.1109/ACCESS.2020.3044858

**Published:** 2020-12-14

**Authors:** Julián D. Arias-Londoño, Jorge A. Gómez-García, Laureano Moro-Velázquez, Juan I. Godino-Llorente

**Affiliations:** 1 Department of Systems EngineeringUniversidad de Antioquia 27983 Medellín 050010 Colombia; 2 Bioengineering and Optoelectronics Laboratory (ByO)Universidad Politécnica de Madrid 16771 28031 Madrid Spain; 3 Department of Electrical and Computer EngineeringJohns Hopkins University 1466 Baltimore MD 21218 USA

**Keywords:** COVID-19, deep learning, pneumonia, radiological imaging, chest X-ray

## Abstract

Current standard protocols used in the clinic for diagnosing COVID-19 include molecular or antigen tests, generally complemented by a plain chest X-Ray. The combined analysis aims to reduce the significant number of false negatives of these tests and provide complementary evidence about the presence and severity of the disease. However, the procedure is not free of errors, and the interpretation of the chest X-Ray is only restricted to radiologists due to its complexity. With the long term goal to provide new evidence for the diagnosis, this paper presents an evaluation of different methods based on a deep neural network. These are the first steps to develop an automatic COVID-19 diagnosis tool using chest X-Ray images to differentiate between controls, pneumonia, or COVID-19 groups. The paper describes the process followed to train a Convolutional Neural Network with a dataset of more than 79, 500 X-Ray images compiled from different sources, including more than 8, 500 COVID-19 examples. Three different experiments following three preprocessing schemes are carried out to evaluate and compare the developed models. The aim is to evaluate how preprocessing the data affects the results and improves its explainability. Likewise, a critical analysis of different variability issues that might compromise the system and its effects is performed. With the employed methodology, a 91.5% classification accuracy is obtained, with an 87.4% average recall for the worst but most explainable experiment, which requires a previous automatic segmentation of the lung region.

## I. Introduction

COVID-19 pandemic has rapidly become one of the biggest health world challenges in recent years. The disease spreads at a fast pace: the reproduction number of COVID-19 ranged from 2.24 to 3.58 during the first months of the pandemic [1], meaning that, on average, an infected person transmitted the disease to 2 or more people. As a result, the number of COVID-19 infections dramatically increased from just a hundred cases in January –most of them concentrated in China– to more than 43 million in November spread all around the world [2].

COVID-19 is caused by the coronavirus SARS-COV2, a virus that belongs to the same family of other respiratory disorders such as the *Severe Acute Respiratory Syndrome* (SARS) and *Middle East Respiratory Syndrome* (MERS). The symptomatology of COVID-19 is diverse and arises after incubation of around 5.2 days. The symptoms might include fever, dry cough, and fatigue; although, headache, hemoptysis, diarrhea, dyspnoea, and lymphopenia are also reported [3], [4]. In severe cases, an *Acute Respiratory Distress Syndrome* (ARDS) might be developed by underlying pneumonia associated with COVID-19. For the most severe cases, the estimated period from the onset of the disease to death ranges from 6 to 41 days (with a median of 14 days), being dependent on the patient’s age and the patient’s immune system status [3].

**FIGURE 1.**
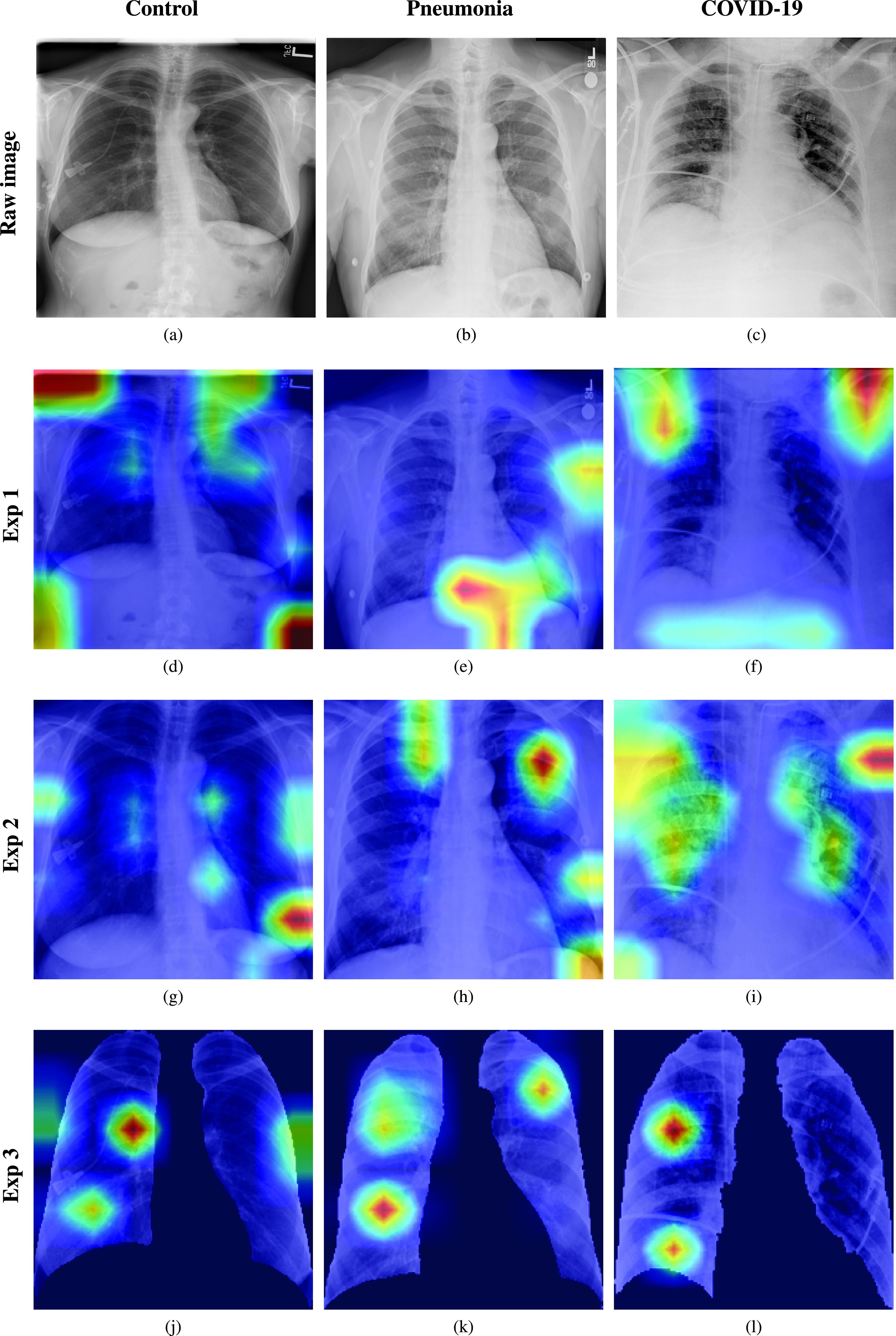
Experiments considered in the paper. First row: raw chest XR images belonging to the control, pneumonia, and COVID-19 classes. Second row: Grad-CAM activation mapping for the XR images. Despite the high accuracy, the model focuses its attention on areas different from the lungs in some cases. Third row: Grad-CAM activation mapping after zooming in, cropping to a squared region of interest and resizing. Zooming to the region of interest forces the model to focus its attention to the lungs, but errors are still present. Fourth row: Grad-CAM activation mapping after a zooming and segmentation procedure. Zooming in and segmenting force the model to focus attention in the lungs. The black background represents the mask introduced by the segmentation procedure.

Once the SARS-COV2 reaches the host’s lung, it gets into the cells through a protein called ACE2, which serves as the “opening” of the cell lock. After the virus’s genetic material has multiplied, the infected cell produces proteins that complement the viral structure to produce new viruses. Then, the virus destroys the infected cell, leaves it, and infects new cells. The destroyed cells produce radiological lesions [5]–[7] such as consolidations and nodules in the lungs, that are observable in the form of ground-glass opacity regions in the *X-Ray* (XR) images (Fig. 1c). These lesions are more noticeable in patients assessed 5 or more days after the onset of the disease, and especially in those older than 50 [8]. Findings also suggest that patients recovered from COVID-19 have developed pulmonary fibrosis [9], in which the connective tissue of the lung gets inflamed, leading to a pathological proliferation of the connective tissue between the alveoli and the surrounding blood vessels. Given these signs, radiological imaging techniques –using plain chest XR and thorax *Computer Tomography* (CT)– have become crucial diagnosis and evaluation tools to identify and assess the severity of the infection.

Since the declaration of the COVID-19 pandemic, the World Health Organization identified four major key areas to reduce the impact of the disease in the world: to prepare and be ready; detect, protect, and treat; reduce transmission; and/or innovate and learn [10]. Concerning the area of detection, significant efforts have been undertaken to improve the diagnostic procedures of COVID-19. To date, the gold standard in the clinic is still a molecular diagnostic test based on a *polymerase chain reaction* (PCR), which is precise but time-consuming, requires specialized personnel and laboratories, and is in general limited by the capacities and resources of the health systems. An alternative to PCR is the rapid tests such as those based on *real-time reverse transcriptase-polymerase chain reaction* (RT-PCR), as they can be more rapidly deployed, decrease the load of the specialized laboratories and personnel, and provide faster diagnosis compared to traditional PCR.

Other tests, such as those based on antigens, are now available but are mainly used for massive testings (i.e. for non-clinical applications) due to a higher chance of missing an active infection. In contrast with RT-PCR, which detects the virus’s genetic material, antigen tests identify specific proteins on the virus’s surface, requiring a higher viral load, which significantly shortens the sensitivity period.

In clinical practice, the RT-PCR test is usually complemented with a chest XR, in such a manner that the combined analysis reduces the significant number of false negatives and, at the same time, brings additional information about the extent and severity of the disease. In addition to that, thorax CT is also used as a second-row method for evaluation. Although the evaluation with CT provides more accurate results in the early stages and have been shown to have greater sensitivity and specificity [11], XR imaging has become the standard in the screening protocols since it is fast, minimally-invasive, low-cost, and requires simpler logistics for its implementation.

In the search for rapid, more objective, accurate and sensitive procedures, which could complement the diagnosis and assessment of the disorder, a trend of research has emerged to employ clinical features extracted from thorax CT or chest XR with automatic detection purposes. A potential benefit of studying the radiological images is that these can characterize pneumonic states even in asymptomatic population [12]. However, more research is needed in this field as the lack of findings in infected patients is also reported [13]. The consolidation of such technology will permit a speedy and accurate diagnosis of COVID-19, decreasing the pressure on microbiological laboratories in charge of the PCR tests and providing more objective means of assessing the disease’s severity. To this end, techniques based on deep learning have been employed to leverage XR information with promising results. Although it would be desirable to employ CT for detection purposes, some significant drawbacks are often present, including higher costs, a more time-consuming procedure, thorough hygienic protocols to avoid infection spread, and the requirement of specialized equipment that might not be readily available in hospitals or health centers. By contrast, XR imaging procedures are available as first screening tests in many hospitals or health centers, at lower expenses.

Several approaches for COVID-19 detection based on chest XR images and different deep learning architectures have been published in the last few months, reporting classification accuracies around 90% or higher. However, the central analysis in most of those works is focused on the variations of network architectures, whereas there is less attention to the variability factors that a real solution should tackle before it can be deployed in the medical setting. In this sense, no analysis has been provided to demonstrate the reliability of the networks’ predictions, which in the context of medical solutions acquires particular relevance. Moreover, most of the works in state of the art have validated their results with data sets containing dozens or a few hundreds of COVID-19 samples, limiting the proposed solutions’ impact.

With these antecedents in mind, this paper uses a deep learning algorithm based on CNN, data augmentation, and regularization techniques to handle data imbalance for the discrimination between COVID-19, controls, and other types of pneumonia. The methods are tested with the most extensive corpus to date, to the authors’ knowledge. Three different sets of experiments were carried out in the search for the most suitable and coherent approach. To this end, the paper also uses explainability techniques to gain insight about the manners on how the neural network learns, and interpretability in terms of the overlapping among the regions of interest selected by the network and those that are more likely affected by COVID-19. A critical analysis of factors that affect the performance of automatic systems based on deep learning is also carried out.

This paper is organized as follows: section II presents some background and antecedents on the use of deep learning for COVID-19 detection. section III presents the methodology, section IV presents the results obtained, whereas V presents the discussions and main conclusions of this paper.

## II. Background

A large body of research has emerged on the use of *Artificial Intelligence* (AI) to detect different respiratory diseases using plain XR images. For instance, in [14] authors developed a 121-layer *Convolutional Neural Network* (CNN) architecture, called Chexnet, which was trained with a dataset of 100, 000 XR images for the detection of different types of pneumonia. The study reports an area under the *Receiving Operating Characteristic* (ROC) curve of 0.76 in a multiclass scenario composed of 14 classes.

Directly related to the COVID-19 detection, three CNN architectures (ResNet50, InceptionV3 and InceptionResNetV2) were considered in [15], using a database of just 50 controls and 50 COVID-19 patients. The best accuracy (98%) was obtained with ResNet50. In [16], seven different deep CNN models were tested using a corpus of 50 controls and 25 COVID-19 patients. The best results were attained with the VGG19 and DenseNet models, obtaining F1-scores of 0.89 and 0.91 for controls and patients. The COVID-Net architecture was proposed in [17]. The net was trained with an open repository, called COVIDx, composed of 13, 975 XR images, although only 358 -from 266 patients– belonged to the COVID-19 class. The attained accuracy was of 93.3%. In [18] a deep anomaly detection algorithm was employed for the detection of COVID-19, in a corpus of 100 COVID-19 images (taken from 70 patients), and 1, 431 control images (taken from 1008 patients). 96% of sensitivity and 70% of specificity was obtained. In [19], a combination of a CNN for feature extraction and a *Long Short Term Memory Network* (LSTM) for classification were used for automatic detection purposes. The model was trained with a corpus gathered from different sources, consisting of 4, 575 XR images: 1, 525 of COVID-19 (although 912 come from a repository applying data augmentation), 1, 525 of pneumonia, and 1, 525 of controls. In a 5-folds cross-validation scheme, a 99% accuracy was reported. In [20], the VGG16 network was used for classification, employing a database of 132 COVID-19, 132 controls and 132 pneumonia images. Following a hold-out validation, about 100% accuracy was obtained identifying COVID-19, being lower on the other classes.

Authors in [21] adapted a model for the classification of COVID-19 by using transfer-learning based on the Xception network. Experiments were carried out in a database of 127 COVID-19, 500 controls, and 500 patients with pneumonia gathered from different sources, attaining about 97% accuracy. A similar approach, followed in [22], used the same corpus for the binary classification of COVID-19 and controls; and for the multiclass classification of COVID-19, controls, and pneumonia. With a modification of the Darknet model for transfer-learning and 5-folds cross-validation, 98% accuracy in binary classification and 87% in multiclass classification was obtained. Another Xception transfer-learning-based approach was presented in [23], but considering two multi-class classification tasks: i) controls vs. COVID-19 vs. viral pneumonia and bacterial pneumonia; ii) controls vs. COVID-19 vs. pneumonia. To deal with the imbalance of the corpus, an undersampling technique was used to randomly discard registers from the larger classes, obtaining 290 COVID-19, 310 controls, 330 bacterial pneumonia, and 327 viral pneumonia chest XR images. The reported accuracy was 89% in the 4-class problem and 94% in the 3-class scenario. Moreover, in a 3-class cross-database experiment, the accuracy was 90%. In [24], four CNN networks (ResNet18, ResNet50, SqueezeNet, and DenseNet-121) were used for transfer learning. Experiments were performed on a database of 184 COVID-19 and 5, 000 no-finding and pneumonia images. Reported results indicate a sensitivity of about 98% and a specificity of 93%. In [25], five state-of-the-art CNN systems –VGG19, MobileNetV2, Inception, Xception, InceptionResNetV2– were tested on a transfer-learning setting to identify COVID-19 from control and pneumonia images. Experiments were carried out in two partitions: one of 224 COVID-19, 700 bacterial pneumonia, and 504 control images; and another that considered the previous normal and COVID-19 data but included 714 cases of bacterial and viral pneumonia. The MobileNetV2 net attained the best results with 96% and 94% accuracy in the 2 and 3-classes classification. In [26], the MobileNetV2 net was trained from scratch and compared to one net based on transfer-learning and to another based on hybrid feature extraction with fine-tuning. Experiments performed in a dataset of 3905 XR images of 6 diseases indicated that training from scratch outperforms the other approaches, attaining 87% accuracy in the multiclass classification and 99% in the detection of COVID-19. A system, also grounded on the InceptionNet and transfer-learning, was presented in [27]. Experiments were performed on 6 partitions of XR images with COVID-19, pneumonia, tuberculosis, and controls. Reported results indicate 99% accuracy, in a 10-folds cross-validation scheme, in the classification of COVID-19 from other classes.

In [28], fuzzy color techniques were used as a pre-processing stage to remove noise and enhance XR images in a 3-class classification setting (COVID-19, pneumonia, and controls). The pre-processed images and the original ones were stacked. Then, two CNN models were used to extract features: MobileNetV2 and SqueezeNet. A feature selection technique based on social mimic optimization and a Support Vector Machine (SVM) was used. Experiments were performed on a corpus of 295 COVID-19, 65 controls and 98 pneumonia XR images, attaining about 99% accuracy.

Given the limited amount of COVID-19 images, some approaches have focused on generating artificial data to train better models. In [29], an auxiliary *Generative Adversarial Network* (GAN) was used to produce artificial COVID-19 XR images from a database of 403 COVID-19 and 1, 124 controls. Results indicated that data augmentation increased accuracy from 85% to 95% on the VGG16 net. Similarly, in [30], GAN was used to augment a database of 307 images belonging to four classes: controls, COVID-19, bacterial and viral pneumonia. Different CNN models were tested in a transfer-learning-based setting, including Alexnet, Googlenet, and Restnet18. The best results were obtained with Googlenet, achieving 99% in a multiclass classification approach. In [31], a CNN based on capsule networks (CapsNet), was used for binary (COVID-19 vs. controls) and multi-class classification (COVID-19 vs. pneumonia vs. controls). Experiments were performed on a dataset of 231 COVID-19, 1, 050 pneumonia and 1, 050 controls XR images. Data augmentation was used to increase the number of COVID-19 images to 1, 050. On a 10-folds cross-validation scheme, 97% accuracy for binary classification, and 84% multi-class classification were achieved. The CovXNet architecture, based on depth-wise dilated convolution networks, was proposed in [32]. In the first stage, pneumonia (viral and bacterial) and control images were employed for pretraining. Then, a a refined model of COVID-19 is obtained using transfer learning. In experiments using two-databases, 97% accuracy was achieved for COVID-19 vs. controls, and of 90% for COVID-19 vs. controls vs. bacterial and viral cases of pneumonia. In [33], an easy-to-train neural network with a limited number of training parameters was presented. To this end, patch phenomena found on XR images were studied (bilateral involvement, peripheral distribution, and ground-glass opacification) to develop a lung segmentation and a patch-based neural network that distinguished COVID-19 from controls. The basis of the system was the ResNet18 network. Saliency maps were also used to produce interpretable results. In experiments performed on a database of controls (191), bacterial pneumonia (54), tuberculosis (57) and viral pneumonia (20), about 89% accuracy was obtained. Likewise, interpretable results were reported in terms of large correlations between the saliency maps’ activation zones and the radiological findings found in the XR images. The authors also indicate that when the lung segmentation approach was not considered, the system’s accuracy decreased to about 80%. In [34], 2D curvelets transformations were used to extract features from XR images. A feature selection algorithm based on meta-heuristic was used to find the most relevant characteristics, while a CNN model based on EfficientNet-B0 was used for classification. Experiments were carried out in a database of 1, 341 controls, 219 COVID-19, and 1, 345 viral pneumonia images, and 99% classification accuracy was achieved with the proposed approach. Multiclass and hierarchical classification of different types of diseases producing pneumonia (with 7 labels and 14 label paths), including COVID-19, were explored in [35]. Since the database of 1, 144 XR images was heavily imbalanced, different resampling techniques were considered. By following a transfer-learning approach based on a CNN architecture to extract features, and a hold-out validation with 5 different classification techniques, a macro-avg F1-Score of 0.65 and an F1-Score of 0.89 were obtained for the multiclass and hierarchical classification scenarios, respectively. In [36], a three-phases approach is presented: i) to detect the presence of pneumonia; ii) to classify between COVID-19 and pneumonia; and, iii) to highlight regions of interest of XR images. The proposed system utilized a database of 250 images of COVID-19 patients, 2, 753 with other pulmonary diseases, and 3, 520 controls. By using a transfer-learning system based on VGG16, about 0.97 accuracy was reported. A CNN-hierarchical approach using decision trees (based on ResNet18) was presented in [37], on which a first tree classified XR images into the normal or pathological classes; the second identified tuberculosis; and the third COVID-19. Experiments were carried out on 3 partitions obtained after having gathered images from different sources and data augmentation. The accuracy for each decision tree–starting from the first– was about 98%, 80%, and 95%, respectively.

### A. Issues Affecting Results in the Literature

Table 1 presents a summary of state of the art in the automatic detection of COVID-19 based on XR images and deep learning. Despite the excellent results reported, the review reveals that some of the proposed systems suffer from certain shortcomings that affect the conclusions extracted in their respective studies, limiting the translational possibilities to the clinical environment. Likewise, variability factors have not been deeply studied in these papers and their study can be regarded as necessary.

**TABLE 1:** Summary of the Literature in the Field

Ref.	Architecture	Number of cases			Classes	Performance metrics	Lung segment.	Explainable
		COVID-19	Controls	Others				
[15]	InceptionV3, InceptionResNetV2, ResNet50	50	50	–	2	Acc=98%	N	N
[16]	VGG19, DenseNet	25	50	–	2	AvF1=0.90	N	N
[17]	COVID-Net, ResNet50, VGG19	358	8066	5538	3	Acc=93.3%	N	N
[18]	EfficientNet	100	1431	–	2	Se=96% Sp=70%	N	N
[19]	CNN + LSTM	1525^*^	1525	1525	3	Acc=99%	N	N
[21]	Xception	127	500	500	3	Acc=97%	N	N
[22]	Darknet	127	500	500	3	Acc=87%	N	N
[23]	Xception	290	310	657	3	Acc=93%	N	N
[25]	VGG19, MobileNetV2, Inception, Xception, InceptionResNetV2	224	504	700	3	Acc=94%	N	N
[28]	MobileNetV2, SqueezeNet	295	65	98	3	Acc=99%	N	N
[27]	Inception	162	2003	4650	3	Acc=99%	N	N
[35]	Inception-V3	90	1000	687	7	AvF1=0.65	N	N
[29]	VGG16	403	1124	–	2	Acc=95%	N	N
[30]	Alexnet, Googlenet, Restnet18	69	79	158	4	Acc=99%	N	N
[31]	Capsnet	231	1050	1050	3	Acc=84%	N	N
[32]	CovXNet	305	305	610	4	Acc=90.2%	N	Y
[33]	ResNet18	180	191	131	4	Acc=89%	Y	Y
[20]	VGG16	132	132	132	3	AvF1=0.85	N	N
[37]	ResNet18	162	585	585	3	Acc=95%	N	N
[24]	ResNet18, ResNet50, SqueezeNet, DenseNet121	184	2400	2600	2	Se=98% Sp=92.9%	N	N
[36]	VGG16	250	3520	2753	4	Acc=97%	N	N
[34]	EfficientNet-B	219	1341	1345	3	Acc=99%	N	N

^*^ 912 coming from a repository of data augmented images.

For instance, one of the issues that affect most of the reviewed systems to detect COVID-19 from plain chest XR images is the use of very limited datasets, which compromises their generalization capabilities.

Indeed, to date and from the authors’ knowledge, the paper employing the largest database of COVID-19 considers 1, 525 XR images gathered from different sources. However, 912 images belong to a data augmented repository, which does not include additional information about the initial number of files or the number of augmented images. In general terms, most of the works employ less than 300 COVID-19 XR images, having systems that use as few as 50 images. However, this is understandable given that some of these works were published during the onset of the pandemics when the number of available registers was limited.

On the other hand, a good balance in the patients’ age is considered essential to avoid the model to learn age-specific features. However, several previous works have used XR images from children to populate the pneumonia class.^1^ This might be biasing the results given the age differences of COVID-19 patients.^1^First efforts used the RSNA Pneumonia Detection Challenge dataset, which is focused on the detection of pneumonia cases in children. https://www.kaggle.com/c/rsna-pneumonia-detection-challenge/overview

Despite many works in the literature report a good performance in detecting COVID-19, most of the approaches follow a brute force approach exploiting deep learning’s potentiality to correlate with the outputs (i.e., the class labels) but provide low interpretability and explainability of the process. It is unclear if the good results are due to the system’s actual capability to extract information related to the pathology or because it leart other aspects during training that are biasing and compromising the results. As a matter of example, just one of the studies reported in the literature follows a strategy that forces the network to focus on the most significant areas of interest for COVID-19 detection [33]. It does so by proposing a methodology based on semantic segmentation of the lungs. In the remaining cases, it is unclear if the models are analyzing the lungs or if they are categorizing given any other information available, which might be interesting for classification purposes but might lack diagnostic interest. This is relevant, as in all the analyzed works in literature, pneumonia and controls classes come from a certain repository, whereas others such as COVID-19 comes from a combination of sources and repositories. Having classes generated in different conditions might undoubtedly affect the results, and as such, a critical study about this aspect is needed. In the same line, other variability issues such as the sensor technology employed, the type of projection used, the sex of the patients, and even age, require a thorough study.

Finally, the literature review revealed that most of the published papers showed excellent correlation with the disease but low interpretability and explainability (see Table 1). Indeed, it is often more desirable in clinical practice to obtain interpretable results that correlate with pathological conditions or a particular demographic or physiological variable than a black box system that yields a binary or a multiclass decision. From the revision of literature, only [33] and [32] partially addressed this aspect. Thus, further research on this topic is needed.

With these ideas in mind, this paper addresses these aspects by training and testing with a wide corpus of RX images, proposing and comparing two strategies to preprocess the images, analyze the effect of some variability factors, and provide some insights to more explainable and interpretable results. The primary goal is to present a critical overview of these aspects since they might be affecting the modeling capabilities of the deep learning systems for the detection of COVID-19.

## III. Methodology

The design methodology is presented in the following section. The procedure followed to train the neural network is described first, along with the process that was followed to create the dataset. The network and the source code to train it are available at https://github.com/jdariasl/COVIDNET, so results can be readily reproduced by other researchers.

### A. The Network

The core of the system is a deep CNN based on the COVID-Net^2^ proposed in [17]. Some modifications were made to include regularization components in the last two dense layers and a weighted categorical cross-entropy loss function to compensate the class imbalance. The network structure was also refactored to allow gradient-based localization estimations [38], which are used after training in the search for an explainable model.^2^Following the PyTorch implementation available at https://github.com/IliasPap/COVIDNet

The network was trained with the corpus described in III-B using the Adam optimizer with a learning rate policy: the learning rate decreases when learning stagnates for some time (i.e., ’patience’). The following hyperparameters were used for training: learning rate = 2^−5^, number of epochs = 24, batch size = 32, factor = 0.5, patience = 3. Furthermore, data augmentation for pneumonia and COVID-19 classes was leveraged with the following augmentation types: horizontal flip, Gaussian noise with a variance of 0.015, rotation, elastic deformation, and scaling. The variant of the COVID-Net was built and evaluated using the PyTorch library [39]. The CNN features from each image are concatenated by a flatten operation, and the resulting feature map is fed to three fully connected layers to generate a probability score for each class. The first two fully connected layers include dropout regularization of 0.3 and ReLU activation functions. Dropout was necessary because the original network tended to overfit since the very beginning of the training phase.

The network’s input layer rescales the images keeping the aspect ratio, with the shortest dimension scaled to 224 pixels. Then, the input image is cropped to a square of $224 \times 224$ pixels located in the center of the image. Images are normalized using a z-score function with parameters $mean=[{0.485, 0.456, 0.406}]$ and $std=[{0.229, 0.224, 0.225}]$, for each of the three RGB channels respectively. Even though we are working with grayscale images, the network architecture was designed to be pre-trained on a general-purpose database including colored images; this characteristic was kept in case it would be necessary to use some transfer learning strategy in the future.

The network’s output layer provides a score for each of the three classes (i.e. control, pneumonia, or COVID-19), which is converted into three probability estimates–in the range [0, 1]– using a softmax activation function. The class membership’s final decision is made according to the highest of the three probability estimates obtained.

### B. The Corpus

The corpora used in the paper have been compiled from a set of *Posterior-Anterior* (PA) and *Anterior-Posterior* (AP) XR images from different public sources. The compilation contains images from participants without any observable pathology (controls or no findings), pneumonia, and COVID-19 cases. After the compilation, two subsets of images were generated, i.e., training and testing. Table 2 contains the number of images per subset and class. Overall, the corpus contains more than 70, 000 XR images, including more than 8, 500 images belonging to COVID-19 patients.

**TABLE 2:** Number of Images per Class for Training and Testing Subsets

Subset	Control	Pneumonia	COVID-19
Training	45022	21707	7716
Testing	4961	2407	857

The repositories of XR images employed to create the corpus used in this paper are presented next. Most of these contain solely registers of controls and pneumonia patients. Only the most recent repositories include samples of COVID-19 XR images. In all cases, the annotations were made by a specialist as indicated by the authors of the repositories.

The COVID-19 class is modelled compiling images coming from three open data collection initiatives: HM Hospitales COVID [40], BIMCV-COVID19 [41] and Actualmed COVID-19 [42] chest XR datasets. The final result of the compilation process is a subset of 8, 573 images from more than 3, 600 patients at different stages of the disease.^3^^3^Figures at the time the datasets were downloaded. The datasets are still open, and more data might be available in the next future.

Table 3 summarizes the most significant characteristics of the datasets used to create the corpus, which is presented next:

**TABLE 3:** Demographic Data of the Datasets Used. Only Those Labels Confirmed are Reported

	Mean age ± std	# Males/# Females	# Images	AP/PA	DX/CR	COVID-19	Pneumonia	Control
HM Hospitales	67,8 ± 15,7	3703/1857 ^*^	5560	5018/542	1264/4296	Y	N	N
BIMCV	62,4 ± 16,7	1527/1486 ^**^	3013	1171/1217	1145/1868	Y	N	N
ACT	–	–	188	30/155	126/59	Y	N	Y
ChinaSet	35,4 ± 14,8	449/213	662	0/662	662/0	N	Y	Y
Montgomery	51,9 ± 2,41	63/74	138	0/138	0/138	N	Y	Y
CRX8	45,75 ± 16,83	34760/27030	61790	21860/39930	61790/0	N	Y	Y
CheXpert	62.38 ± 18,62	2697/1926	4623	3432/1191	–	N	Y	N
MIMIC	–	–	16399	10850/5549	–	N	Y	N

^*^ 1377/929 patients

^**^ 727/626 patients

#### 1) HM Hospitales COVID-19 Dataset

This dataset was compiled by HM Hospitals [40]. It contains all the available clinical information about anonymous patients with the SARS-CoV-2 virus treated in different centers belonging to this company since the beginning of the pandemic in Madrid, Spain.

The corpus contains the anonymized records of 2, 310 patients and includes several radiological studies for each patient corresponding to different stages of the disease. A total of 5, 560 RX images are available in the dataset, with an average of 2.4 image studies per subject, often taken in intervals of two or more days. The histogram of the patients’ age is highly coherent with the demographics of COVID-19 in Spain (see Table 3 for more details).

Only patients with at least one positive PCR test or positive immunological tests for SARS-CoV-2 were included in the study. The Data Science Commission and the Research Ethics Committee of HM Hospitales approved the current research study and the data for this purpose.

#### 2) BIMCV COVID19 Dataset

BIMCV COVID19 dataset [41] is a large dataset with chest radiological studies (XR and CT) of COVID-19 patients along with their pathologies, results of PCR and immunological tests, and radiological reports. It was recorded by the Valencian Region Medical Image Bank (BIMCV) in Spain. The dataset contains the anonymized studies of patients with at least one positive PCR test or positive immunological tests for SARS-CoV-2 between February 26th and April 18th, 2020. The corpus is composed of 3, 013 XR images, with an average of 1.9 image studies per subject, taken in intervals of approximately two or more days. The histogram of the patients’ age is highly coherent with the demographics of COVID-19 in Spain (Table 3). Only patients with at least one positive PCR test or positive immunological tests for SARS-Cov-2 were included in the study.

#### 3) Actualmed Set (ACT)

The actualmed COVID-19 Chest XR dataset initiative [42] contains a series of XR images compiled by Actualmed and Universitat Jaume I (Spain). The dataset contains COVID-19 and control XR images, but no information is given about the place or date of recording and/or demographics. However, a metadata file is included. It contains an anonymized descriptor to distinguish among patients and information about the XR modality, type of view, and the class to which the image belongs.

#### 4) China Set - the Shenzhen Set

The set was created by the National Library of Medicine, Maryland, USA, in collaboration with the Shenzhen No.3 People’s Hospital at Guangdong Medical College in Shenzhen, China [43].

The dataset contains normal and abnormal chest XR with manifestations of tuberculosis and includes associated radiologist readings.

#### 5) The Montgomery Set

The National Library of Medicine created this dataset in collaboration with the Department of Health and Human Services, Montgomery County, Maryland, USA. It contains data from XR images collected under Montgomery County’s tuberculosis screening program [43], [44].

#### 6) ChestX-ray8 Dataset (CRX8)

The ChestX-ray8 dataset [45] contains 12, 120 images from 14 common thorax disease categories from 30, 805 unique patients, compiled by the National Institute of Health (NIH). For this study, the images labeled with ’no radiological findings’ were used to be part of the control class, whereas the images annotated as ’pneumonia’ were used for the pneumonia class.

#### 7) CheXpert Dataset

CheXpert [46] is a dataset of XR images created for an automated evaluation of medical imaging competitions and contains chest XR examinations carried out in Stanford Hospital during 15 years. For this study, we selected 4, 623 pneumonia images using those annotated as ’pneumonia’ with and without additional comorbidity. COVID-19 never caused these comorbidities. The motivation to include pneumonia with comorbidities was to increase the number of pneumonia examples in the final compilation for this study, increasing this cluster’s variability.

#### 8) MIMIC-CXR Database

MIMIC-CXR [47] is an open dataset complied from 2011 to 2016, and comprising de-identified chest RX from patients admitted to the Beth Israel Deaconess Medical Center. In our study, we employed the images for the pneumonia class. The labels were obtained from the agreement of the two methods indicated in [47]. The dataset reports no information about gender or age; thus, we assume that the demographics are similar to those of CheXpert dataset and those of pneumonia [48].

### C. Image Pre-Processing

XR images were converted to uncompressed grayscale ’.png’ files, encoded with 16 bits, and preprocessed using the DICOM *WindowCenter* and *WindowWidth* details (when needed). All images were converted to a *Monochrome 2* photometric interpretation. Initially, the images were not re-scaled to avoid loss of resolution in later processing stages.

Only AP and PA views were selected. No differentiation was made between erect, either standing or sitting, or decubitus. This information was inferred by a careful analysis of the DICOM tags and required manual checking due to certain labeling errors.

### D. Experiments

The corpus collected from the aforementioned databases was processed to compile three different datasets of equal size to the initial one. Each of these datasets was used to run a different set of experiments.

#### 1) Experiment 1. Raw Data

The first experiment was run using the raw data extracted from the different datasets. Each image is kept with the original aspect ratio. Only a histogram equalization was applied.

#### 2) Experiment 2. Cropped Image

**FIGURE 2.**
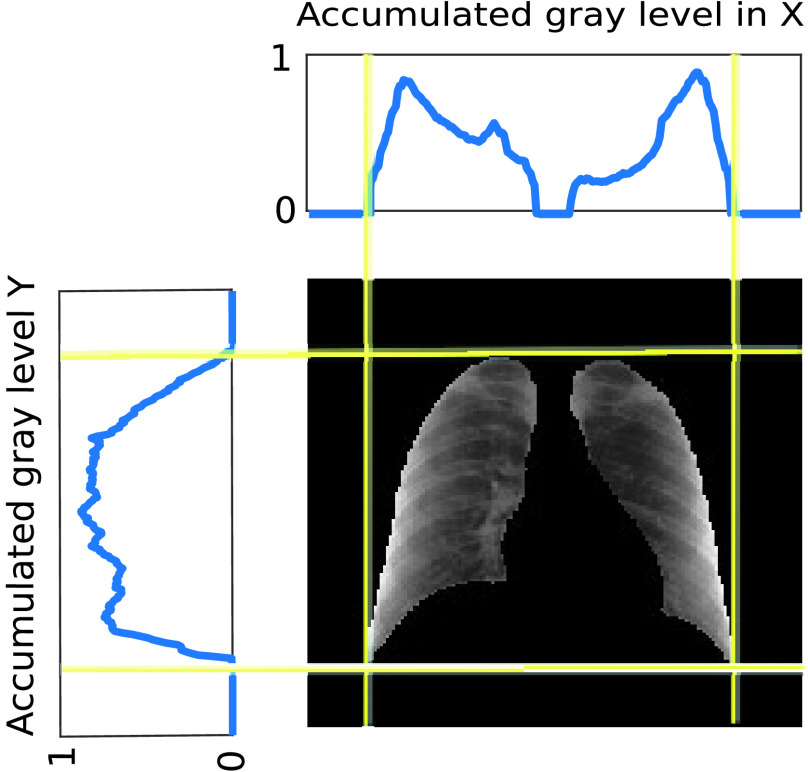
Identification of the squared region of interest. Plots in the top and left represent the normalized accumulated gray level in the vertical and horizontal dimension respectively.

The second experiment consists of preprocessing the images by zooming in, cropping to a squared region of interest, and resizing to a squared image (aspect ratio 1:1). The process is summarized in the following steps:

1) Lungs are segmented from the original image using a U-Net semantic segmentation algorithm.^4^ The algorithm used reports *Intersection-Over-Union* (IoU) and Dice similarity coefficient scores of 0.971 and 0.985 respectively.
2) A black mask is extracted to identify the external boundaries of the lungs.
3) The mask is used to create two sequences, adding the grey levels of the rows and columns respectively. These two sequences provide four boundary points, which define two segments of different lengths in the horizontal and vertical dimensions.
4) The sequences of added grey levels in the vertical and horizontal dimensions of the mask are used to identify a squared region of interest associated with the lungs, taking advantage of the higher added values outside the lungs (Fig. 2). The process to obtain the squared region requires identifying the middle point of each of the identified segments and cropping in both dimensions using the length of the longest of these two segments.
5) The original image is cropped with a squared template placed in the centre of the matrix using the information obtained in the previous step. No mask is placed over the image.
6) Histogram equalization of the image obtained.

^4^Following the Keras implementation available at https://github.com/imlab-uiip/lung-segmentation-2d

This process is carried out to decrease the variability of the data, to make the training process of the network simpler, and to ensure that the region of significant interest is in the centre of the image with no areas cut.

#### 3) Experiment 3. Lung Segmentation

The third experiment consists of preprocessing the images by masking, zooming in, cropping to a squared region of interest, and resizing to a squared image (aspect ratio 1:1). The process is summarized in the following steps:

1) Lungs are segmented from the original image using the same semantic segmentation algorithm used in experiment 2.
2) An external black mask is extracted to identify the external boundaries of the lungs.
3) The mask is used to create two sequences, adding the grey levels of the rows and columns respectively.
4) The sequences of added grey levels in the vertical and horizontal dimensions of the mask are used to identify a squared region of interest associated to the lungs, taking advantage of the higher added values outside them (Fig. 2).
5) The original image is cropped with a squared template placed in the center of the image.
6) The mask is dilated with a $5 \times 5$ pixels kernel, and it is superimposed to the image.
7) Histogram equalization is applied only to the segmented area (i.e. the area corresponding to the lungs).

This preprocessing makes the training of the network much simpler and forces the network to focus the attention on the lungs region, removing external characteristics –like the sternum– that might influence the obtained results.

### E. Identification of the Areas of Significant Interest for the Classification

The areas of significant interest used by the CNN for discrimination purposes are identified using a qualitative analysis based on a *Gradient-weighted Class Activation Mapping* (Grad-CAM) [38]. This is an explainability method that serves to provide insights about the manners on how deep neural networks learn, pointing to the most significant areas of interest for decision-making purposes. The method uses the gradients of any target class to flow until the final convolutional layer, and to produce a coarse localization map which highlights the most important regions in the image identifying the class. The result of this method is a heat map like those presented in Fig. 1, in which the colour encodes the importance of each pixel in differentiating among classes.

## IV. Results

**FIGURE 3.**
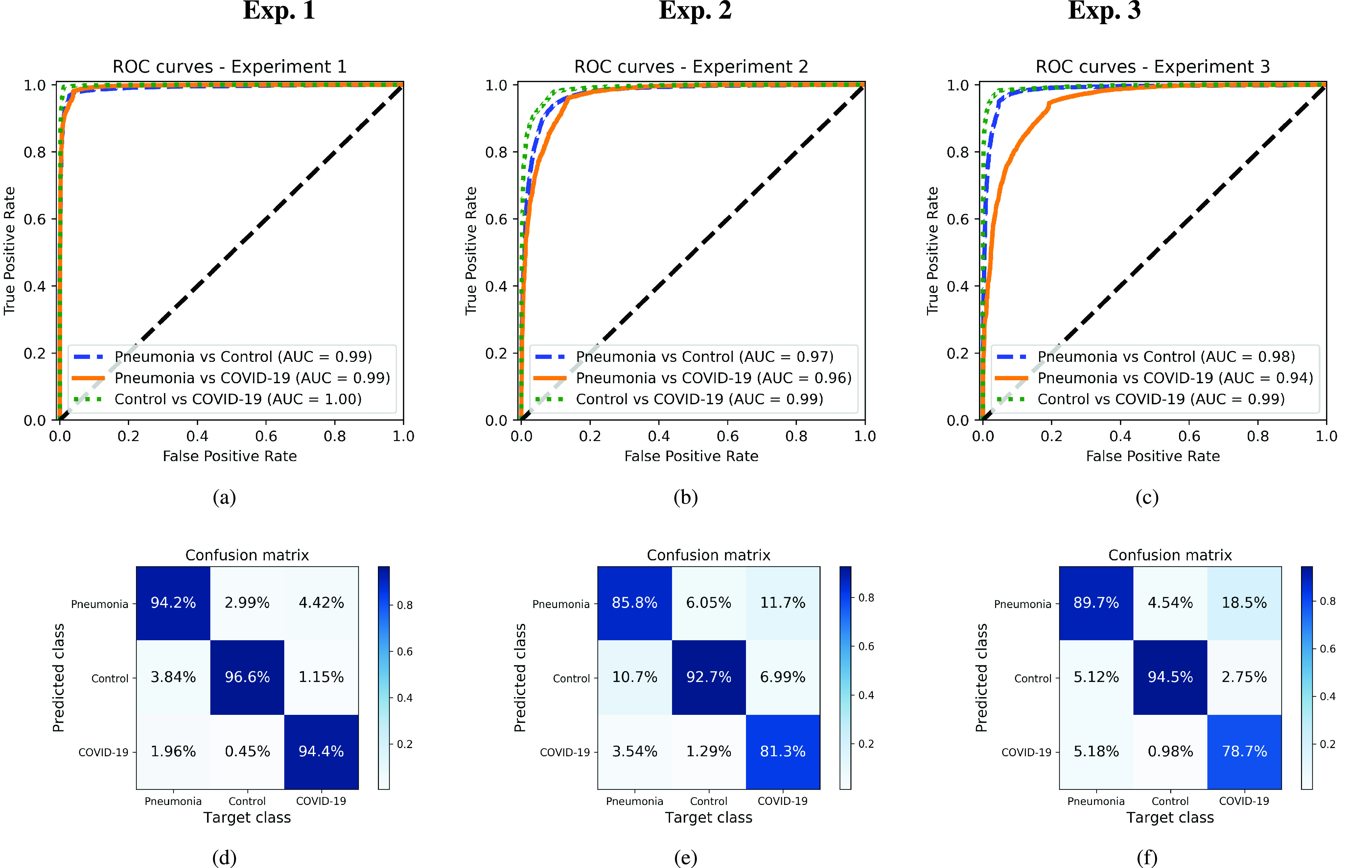
ROC curves and confusion matrices for each one of the experiments, considering each one of the classes separately. Top: ROC curves. Bottom: Normalized confusion matrices. Left: Original images (experiment 1). Center: Cropped Images (experiment 2). Right: Segmented images (experiment 3).

**FIGURE 4.**
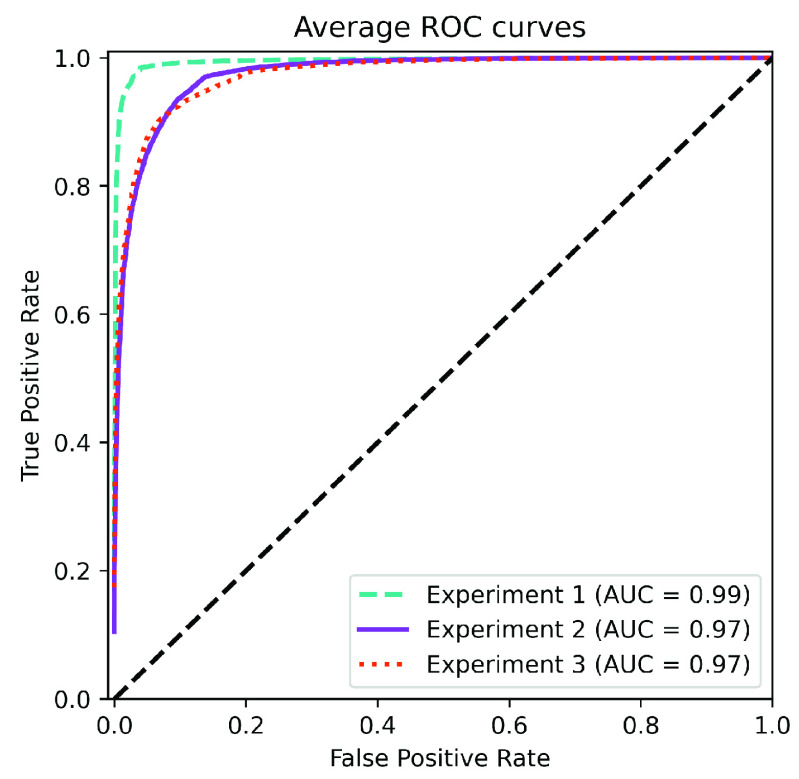
Average ROC curves for each experiment, including AUC values.

The model has been quantitatively evaluated computing the test *Positive Predictive Value* (PPV), *Recall*, *F1-score* (F1), *Accuracy* (Acc), *Balanced Accuracy* (BAcc), *Geometric Mean Recall* (GMR) and *Area Under the ROC Curve* (AUC) for each of the three classes in the corpus previously described in section III-B. The performance of the models is assessed using an independent testing set, which has not been used during development. A 5-folds cross-validation procedure has been used to evaluate the obtained results (Training/Test balance: 90/10 %). The performance of the CNN network on the three experiments considered in this paper is summarized in Table 4. Likewise, the ROC curves per class for each of the experiments, and the corresponding confusion matrices are presented in Fig. 3. The global ROC curve displayed in Fig. 4 for each experiment summarizes the global performance of the experiments.

**TABLE 4:** Performance Measures for the Three Experiments Considered in the Paper

Experiment	Class	Measures					
		PPV	Recall	F1	Acc	BAcc	GMR
Exp. 1	Pneumonia	92.53 ± 1.13	94.20 ± 1.43	93.35 ± 0.68	91.67 ± 2.56	94.43 ± 1.36	93.00 ± 1.00
	Control	93.35 ± 0.68	96.56 ± 0.50	97.24 ± 0.23			
	COVID-19	91.67 ± 2.56	94.43 ± 1.36	93.00 ± 1.00			
Exp. 2	Pneumonia	84.02 ± 1.16	85.75 ± 1.46	84.86 ± 0.51	87.64 ± 0.74	81.35 ± 2.70	81.36 ± 0.42
	Control	93.62 ± 0.76	92.67 ± 0.69	93.14 ± 0.25			
	COVID-19	81.60 ± 3.33	81.35 ± 2.70	81.36 ± 0.42			
Exp. 3	Pneumonia	85.26 ± 0.73	85.26 ± 0.73	87.42 ± 0.27	91.53 ± 0.20	87.64 ± 0.74	87.37 ± 0.84
	Control	96.99 ± 0.17	94.48 ± 0.24	95.72 ± 0.15			
	COVID-19	78.52 ± 2.08	78.73 ± 2.80	78.57 ± 1.15

Considering experiment 1, and although slightly higher for controls, the detection performance remains almost similar for all classes (the PPV ranges from 91–93%) (Table 4). The remaining measures per class follow the same trend, with similar figures but better numbers for the controls. ROC curves and confusion matrices of Fig. 3a and Fig. 3d point out that the largest source of confusion for COVID-19 is the pneumonia class. The ROC curves for each one of the classes reach in all cases AUC values larger than 0.99, which, in principle is considered excellent. In terms of global performance, the system achieves an Acc of 91% and a BAcc of 94% (Table 4). This is also supported by the average ROC curve of Fig. 4, which reveals the excellent performance of the network and the almost perfect behaviour of the ROC curve. Deviations are small for the three classes.

When experiment 2 is considered, a decrease in the performance per class is observed in comparison to experiment 1. In this case, the PPV ranges from 81–93% (Table 4), with a similar trend for the remaining figures of merit. ROC curves and confusion matrices in Fig. 3a and Fig. 3d report AUC values in the range 0.96–0.99, and an overlapping of the COVID-19 class mostly with pneumonia. The global performance of the system -presented in the ROC curve of Fig. 4 and Table 4- yields an AUC of 0.98, an Acc of 87% and a BAcc of 81%.

Finally, for experiment 3, PPV ranges from 78% – 96% (Table 4). In this case, the results are slightly worse than those of experiment 2, with the COVID-19 class presenting the worse performance among all the tests. According to Fig. 3c, AUCs range from 0.94 to 0.98. Confusion matrix in Fig. 3f reports a large level of confusion in the COVID-19 class being labelled as pneumonia 18% of the times. In terms of global performance, the system reaches an Acc of 91% and a BAcc of 87% (Table 4). These results are consistent with the average AUC of 0.97 shown in Fig. 4.

### A. Explainability and Interpretability of the Models

The regions of interest identified by the network were analyzed qualitatively using Grad-CAM activation maps [38]. Results shown by the activation maps, permit the identification of the most significant areas in the image, highlighting the zones of interest that the network is using to discriminate.

In this regard, Fig. 1, presents examples of the Grad-CAM of a control, a pneumonia, and a COVID-19 patient, for each of the three experiments considered in the paper. It is important to note that the activation maps are providing overall information about the behaviour of the network, pointing to the most significant areas of interest, but the whole image is supposed to be contributing to the classification process to a certain extent.

The second row in Fig. 1 shows several prototypical results applying the Grad-CAM techniques to experiment 1. The examples show the areas of significant interest for a control, pneumonia and COVID-19 patient.

The results suggest that the detection of pneumonia or COVID-19 is often carried out based on information that is outside the expected area of interest, i.e. the lung area. In the examples provided, the network focuses on the corners of the XR image or in areas around the diaphragm. In part, this is likely due to the metadata which is frequently stamped on the corners of the XR images. The Grad-CAM plots corresponding to the experiment 2 (third row of Fig. 1), indicates that the model still points towards areas which are different from the lungs, but to a lesser extent. Finally, the Grad-CAM of experiment 3 (fourth row of Fig. 1) presents the areas of interest where the segmentation procedure is carried out. In this case, the network is forced to look at the lungs, and therefore this scenario is supposed to be more realistic and more prone to generalizing as artifacts that might bias the results are somehow discarded.

**FIGURE 5.**
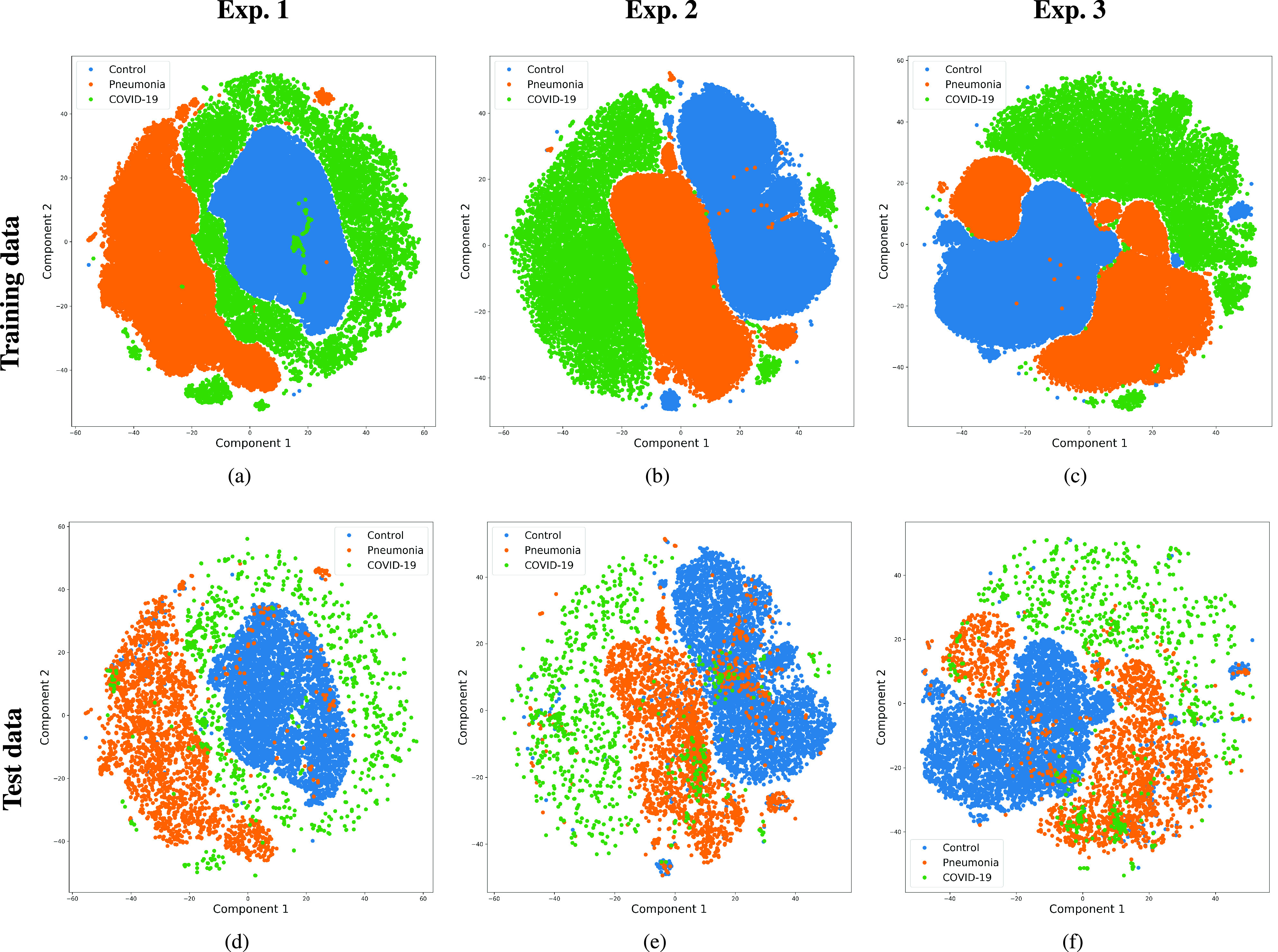
Mapping of the high-dimensional data of the layer adjacent to the output into a two dimensional plot. Top: Output network embedding using t-SNE for the training data. Bottom: Output network embedding using t-SNE for the testing data. Left: Original images (experiment 1). Center: Cropped Images (experiment 2). Right: Segmented images (experiment 3).

On the other hand, for visualization purposes, and in order to interpret the separability capabilities of the system, a t-SNE embedding is used to project the high dimensional data of the layer adjacent to the output of the network, to a 2-dimensional space. Results are presented in Fig. 5 for each of the three experiments considered in the paper.

Fig. 5 indicates that a good separability exists for all the classes in both training and testing data, and for all experiments. The boundaries of the normal cluster are very well defined in the three experiments, whereas pneumonia and COVID-19 are more spread, overlapping with adjacent classes.

In general terms, the t-SNE plots demonstrate the ability of the network to learn a mapping from the input data to the desired labels. However, despite the shape differences found for the three experiments, no additional conclusions can be extracted.

### B. Potential Variability Factors Affecting the System

There are several variability factors which might be biasing the results, namely: the projection (PA vs. AP); the technology of the detector (*Computed Radiography* (CR) vs. *Digital Radiography* (DX)); the gender of the patients; the age; potential specificities of the dataset; or having trained with several images per patient.

The use of several images per patient represents a certain risk of data leak in the COVID-19 class due to its underlying imbalance. However, our initial hypothesis is that using several images per COVID-19 patient but obtained at different instants in time (with days of difference), would increase the variability of the dataset, and thus that source of bias would be disregarded. Indeed, the evolution of the associated lesions often found in COVID-19 is considered fast, in such a manner that very different images are obtained in a time interval as short as one or two days of the evolution. Also, since every single exploration is framed differently, or sometimes even taken with different machines and/or projections, the potential bias is expected to be minimized.

Concerning the type of projection, and to evaluate its effectiveness, the system has been studied taking into account this potential variability factor, which is considered to be one of the most significant. In particular, Table 5, presents the outcomes after accounting for the influence of the XR projection (PA/AP) in the performance of the system. In general terms, the system demonstrates consistency with respect to the projection used, and differences are mainly attributable to smaller training and testing sets. However, significant differences are shown for projection PA in class COVID-19/experiment 3, decreasing the F1 up to 65.61%. The reason for the unexpected drop in performance is unknown, but likely attributable to an underrepresented class in the corpus (see Table 3).

**TABLE 5:** Performance Measures Considering the XR Projection (PA/AP)

Experiment	Class	PA			AP		
		PPV	Recall	F1	PPV	Recall	F1
Exp. 1	Pneumonia	91.25 ± 1.22	92.78 ± 1.58	92.00 ± 0.93	94.70 ± 0.79	96.28 ± 1.10	95.48 ± 0.50
	Control	98.54 ± 0.33	97.83 ± 0.23	98.18 ± 0.14	97.87 ± 0.28	95.46 ± 0.87	96.65 ± 0.43
	COVID-19	84.06 ± 3.94	88.91 ± 2.31	86.33 ± 1.80	95.13 ± 2.46	97.18 ± 0.94	96.12 ± 1.06
Exp. 2	Pneumonia	81.77 ± 1.79	79.17 ± 2.38	80.41 ± 1.16	87.39 ± 1.66	90.78 ± 1.21	89.03 ± 0.71
	Control	94.81 ± 0.46	95.56 ± 0.61	95.33 ± 0.16	92.79 ± 1.53	88.15 ± 1.61	90.38 ± 0.32
	COVID-19	73.72 ± 2.37	68.82 ± 5.20	71.01 ± 2.27	84.96 ± 2.27	87.63 ± 2.04	86.23 ± 0.86
Exp. 3	Pneumonia	84.07 ± 1.72	87.19 ± 1.66	85.57 ± 0.53	87.39 ± 0.97	81.66 ± 1.12	89.47 ± 0.41
	Control	97.88 ± 0.36	97.08± 0.21	97.48± 0.19	96.03 ± 0.81	90.65 ± 0.87	93.26 ± 0.47
	COVID-19	66.68 ± 4.82	65.23 ± 4.73	65.61 ± 1.05	81.82 ± 3.07	83.62 ± 2.14	82.65 ± 1.28

Besides, Table 6 shows –for the three experiments under evaluation and for the COVID-19 class– the error distribution with respect to the sex of the patient, technology of the detector, dataset and projection. For the four variability factors enumerated, results show that the error distribution committed by the system follows –with minor deviations– the existing proportion of the samples in the corpus. These results suggest that there is no clear bias with respect to these potential variability factors, at least for the COVID-19 class which is considered the worst-case due to its underrepresentation. Similar results would be expected for control and pneumonia classes, but these results are not provided due to the lack of certain labels in some of the datasets used (see Table 3).

**TABLE 6:** Percentage of Testing Samples and Error Distribution With Respect to Several Potential Variability Factors for the COVID-19 Class. (% in Hits Represents the Percentage of Samples of Every Factor Under Analysis in the Correctly Predicted Set)

Factor	Types	% in test	% in hits		
			Exp. 1	Exp. 2	Exp. 3
Projection	AP	79	80.0	82.6	82.7
	PA	21	20.0	17.4	17.3
Sensor	DX	22	22.0	23.3	23.6
	CR	78	78.0	76.7	76.4
Sex	M	64	64.0	65.4	65.2
	F	36	36.0	34.6	34.8
DB	BMICV	30	28.7	26.6	26.6
	HM	69	71.0	72.7	73.1
	ACT	1	0.3	0.7	0.3

Concerning age, the datasets used are reasonably well balanced (Table 3), but with a certain bias in the normal class: COVID-19 and pneumonia classes have very similar average ages, but controls have a lower mean age. Our assumption has been that age differences are not significantly affecting the results, but the mentioned difference might explain why the normal cluster in Fig. 5 is less spread than the other two. In any case, no specific age biases have been found in the errors committed by the system.

**FIGURE 6.**
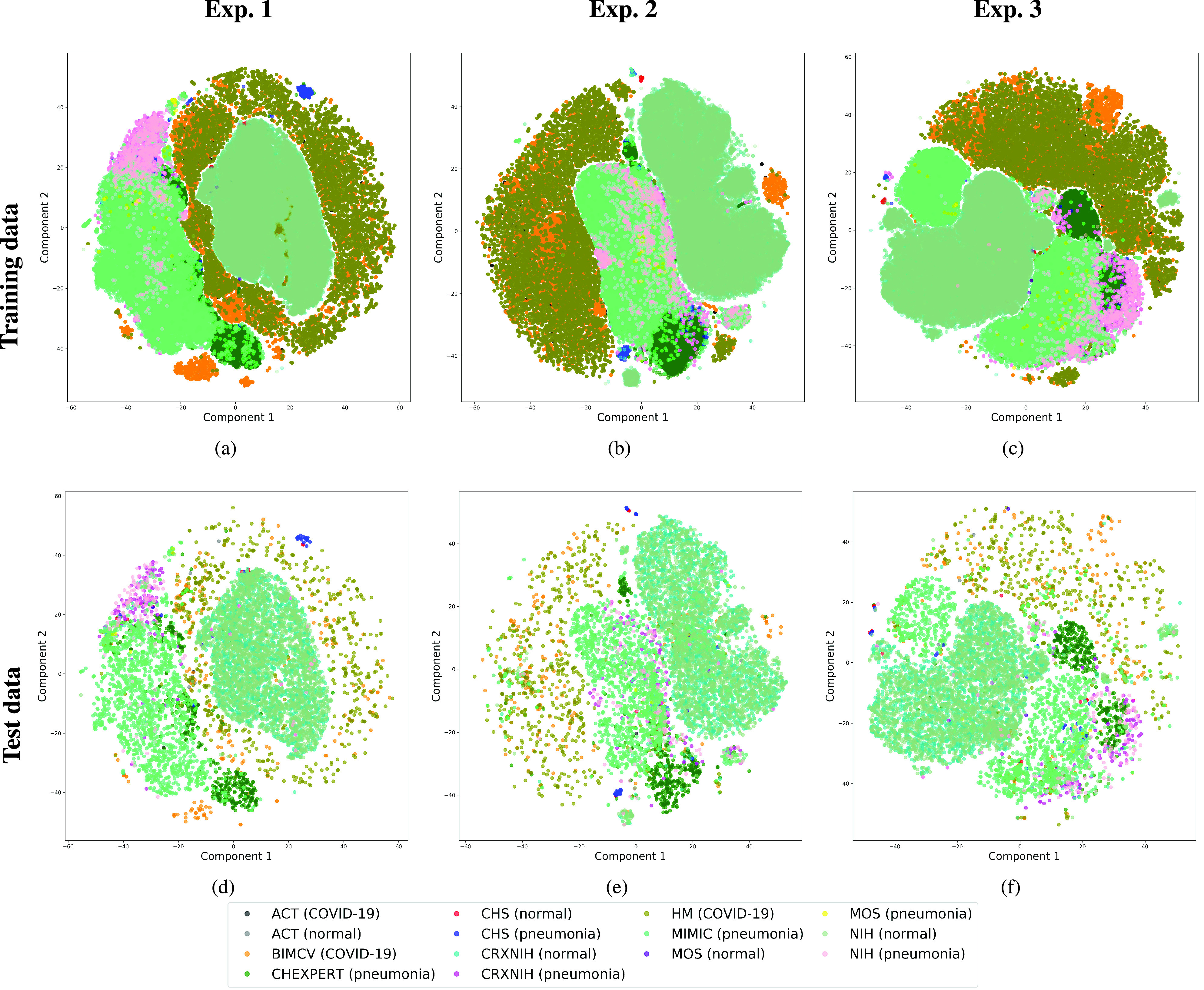
Mapping of the high-dimensional data of the layer adjacent to the output into a two dimensional plot. Top: Output network embedding using t-SNE for the training data. Bottom: Output network embedding using t-SNE for the testing data. Left: Original images (experiment 1). Center: Cropped Images (experiment 2). Right: Segmented images (experiment 3). Labels correspond to data sets and classes.

An additional study was also carried out to evaluate the influence of potential specificities of the different datasets used to compile the corpus (i.e. the variability of the results with respect to the datasets merged to build the corpus). This variability factor is evaluated in Fig. 6 using different t-SNE plots (one for each experiment in a similar way than in Fig. 5) but differentiating the corresponding cluster for each dataset and class.

Results for the different datasets and classes are clearly merged or are adjacent in the same cluster. However, several datasets report a lower variability for certain classes (i.e. variability in terms of scattering). This is especially clear in Chexpert and NIH pneumonia sets, which are successfully merged with the corresponding class but appear clearly clustered, suggesting that these datasets have certain unknown specific characteristics different to those of the complementary datasets. The model has been able to manage this aspect but is a factor to be analyzed in further studies.

## V. Discussion and Conclusion

This study evaluates a deep learning model for the detection of COVID-19 from RX images. The paper provides additional evidence to the state of the art, supporting the potential of deep learning techniques to accurately categorize XR images corresponding to control, pneumonia, and COVID-19 patients (Fig. 1). These three classes were chosen under the assumption that they can support clinicians in making better decisions, establishing potential differential strategies to handle patients depending on their cause of infection [17]. However, the main goal of the paper was not to demonstrate the suitability of deep learning for categorizing XR images but to make a thoughtful evaluation of the results and the different preprocessing approaches, searching for better explainability and interpretability of the results while providing evidence of potential effects that might bias results.

The model relies on the COVID-Net network, which has served as a basis for the developing a more refined architecture. This network has been chosen due to its tailored characteristics and given the previous good results reported by other researchers. The COVID-Net was trained with a corpus compiled using data gathered from different sources: the control and pneumonia classes –with 49, 983 and 24, 114 samples respectively– were collected from the ACT, Chinaset, Montgomery, CRX8, CheXpert, and MIMIC datasets; and the COVID-19 class was collected from the information available at the BIMCV, ACT, and HM Hospitales datasets.

Although the COVID-19 class only contains 8, 573 chest RX images, the developers of the data sources are continuously adding new cases to the respective repositories, so the number of samples is expected to grow in the future. Despite the unbalance of the COVID-19 class, up to date, and to the authors’ knowledge, this is the most extensive compilation of COVID-19, images based on open repositories. Despite that, the number of COVID-19 RX images is still considered small compared to the other two classes. Therefore, it was necessary to compensate for the class imbalance by modifying the network architecture, including regularization components in the last two dense layers. To this end, a weighted categorical cross-entropy loss function was used to compensate for this effect. Likewise, data augmentation techniques were used for pneumonia and COVID-19 classes to generate more samples for these two underrepresented classes automatically.

We stand that automatic diagnosis is much more than a classification exercise, meaning that many factors have to be considered to bring these techniques to clinical practice. In this respect, there is a classic assumption in the literature that the associated heat maps –calculated with Grad-CAM techniques- provide a clinical interpretation of the results, which is unclear in practice. In light of the results shown in the heat maps depicted in Fig. 1, we show that experiment 1 must be carefully interpreted. Despite the high-performance metrics obtained in experiment 1, the significant areas identified by the network are pointing towards certain areas with no clear interest for the diagnosis, such as corners of the images, the sternum, clavicles, etc. From a clinical point of view, this is biasing the results. It means that other approaches are necessary to force the network to focus on the lung area. In this respect, we have developed and compared the results with two preprocessing approaches based on cropping the images and segmenting the lung area (experiment 2 and experiment 3). Again, given the heat maps corresponding to experiment 2, we also see similar explainability problems to those enumerated for experiment 1. The image area reduction proposed in experiment 2 significantly decreases the system’s performance by removing the metadata that usually appears in the top left or right corner. This technique removes areas that can help categorize the images but have no interest from the diagnosis point of view. However, while comparing experiments 2 and 3, performance results improve in the third approach, which focuses on the same region of interest but with a mask that forces the network to see only the lungs. Thus, results obtained in experiments 2 and 3 suggest that eliminating the needless features extracted from the background or non-related regions improves the results. Besides, the third approach (experiment 3) provides more explainable and interpretative results, with the network focusing its attention only on the area of interest for the disease. The gain in explainability of the last method is still at the cost of a lower accuracy with respect to experiment 1, but the improvement in explainability and interpretability is considered critical in translating these techniques to the clinical setting. Despite the decrease in performance, the proposed method in experiment 3 has provided promising results, with an 91.53% Acc, 87.6 BAcc, 87.37% GMR, and 0.97 AUC.

Performance results obtained are in line with those presented in [17], which reports sensitivities of 95, 94, and 91 for control, pneumonia, and COVID-19 classes respectively–also modeling with the COVID-Net in similar conditions as our experiment 1–, but training with a much smaller corpus of 358 RX images from 266 COVID-19 patients, 8, 066 controls, and 5, 538 RX images belonging to patients with different types of pneumonia.

The paper also critically evaluates the effect of several variability factors that might compromise the network’s performance. For instance, the projection (PA/AP) effect was evaluated by retraining the network and checking the outcomes. This effect is important, given that PA projections are often practiced in erect positions to observe pulmonary ways better and are expected to be examined in healthy or slightly affected patients. In contrast, AP projections are often preferred for patients confined in bed, and as such are expected to be practised in the most severe cases. Since AP projections are common in COVID-19 patients, in these cases, more blood will flow to the lungs’ apices than when standing; thus, not considering this variability factor may result in a misdiagnosis of pulmonary congestion [49]. Indeed, the obtained results have highlighted the importance of taking into account this factor when designing the training corpus, as PPV decreases for PA projections in our experiments with COVID-19 images. This issue is probably due to an underrepresentation of this class (Table 5), which would require a further specific analysis when designing future corpora.

On the other hand, results have shown that the error distribution for the COVID-19 class follows a similar proportion to the percentage of images available in the corpus while categorizing by gender, the detector’s technology, the projection, and the dataset. These results suggest no significant bias with respect to these potential variability factors, at least for the COVID-19 class, which is the less represented one.

An analysis of how the clusters of classes were distributed is also presented in Fig. 5, demonstrating how each class is differentiated. These plots help identify existing overlap among classes (especially that present between pneumonia and COVID-19, and to a lesser extent between controls and pneumonia). Similarly, since the corpus used to train the network was built around several datasets, a new set of t-SNE plots was produced, but differentiating according to each of the subsets used for training (Fig. 6). This test served to evaluate the influence of each dataset’s potential specific characteristics in the training procedure and, hence, possible sources of confusion that arise due to particularities of the corpora that are tested. The plots suggest that the different datasets are correctly merged in general terms, but with some exceptions. These exceptions suggest that there might be certain unknown characteristics in the datasets used, which cluster the images belonging to the same dataset together.

The COVID-Net has also demonstrated being a good starting point for the characterization of the disease employing XR images. Indeed, the paper’s outcomes suggest the possibility to automatically identify the lung lesions associated with a COVID-19 infection (see Fig.1) by analyzing the Grad-CAM mappings of experiment 3, providing an explainable justification about the way the network works. However, the interpretation of the heat maps obtained for the control class must be carried out carefully. Whereas the areas of significant interest for pneumonia and COVID-19 classes are supposed to point to potential lesions (i.e. with higher density or with different textures in contrast to controls), the areas of significant interest for the classification in the control group are supposed to correspond to something complementary, potentially highlighting less dense areas. Thus, in the control class, these areas do not point towards any kind of lesion in the lungs.

Likewise, the system developed in experiment 3 attains comparable results to those achieved by a human evaluator differentiating pneumonia from COVID-19. In this respect, the ability of seven radiologists to correctly differentiate pneumonia and COVID-19 from XR images was tested in [50]. The results indicated that the radiologists achieved sensitivities ranging from 97% to 70% (mean 80%), and specificities ranging from 7% to 100% (mean 70%). These results suggest that AI systems have a potential use in a supervised clinical environment.

COVID-19 is still a new disease, and much remains to be studied. The use of deep learning techniques would potentially help understand the mechanisms on how the SARS-CoV2 attacks the lungs and alveoli and how it evolves during the different stages of the disease. Despite there is some empirical evidence on the evolution of COVID-19 –based on observations made by radiologists [6]–, the employment of automatic techniques based on machine learning would help analyze data massively, guide research onto certain paths, or extract conclusions faster. Nevertheless more interpretable and explainable methods are required to go one step forward.

Inline with the previous comment, and based on the empirical evidence respecting the evolution of the disease, it has been stated that during the early stages of the disease, ground-glass shadows, pulmonary consolidation and nodules, and local consolidation in the centre with peripheral ground-glass density are often observed. However, once the disease evolves, the consolidations reduce their density resembling a ground-glass opacity that can derive in a “white lung” if the disease worsens or in a minimization of the opacities if the course of the disease improves [6]. In this manner, if any of these characteristic behaviours are automatically identified, it would be possible to stratify the disorder’s stage according to its severity. Computing the extent of the ground-glass opacities or densities would also be useful to assess the severity of the infection or to evaluate the evolution of the disease. In this regard, the infection extent assessment has been previously tested in other CT studies of COVID-19 [51] using manual procedures based on observation of the images.

Solutions like the one discussed in this paper are intended to support a much faster diagnosis and alleviate radiologists and specialists’ workload, but not to substitute their assessment. A rigorous validation would open the door to integrating these algorithms in desktop applications or cloud servers for its use in the clinic environment. Thus, its use, maintenance, and update would be cost-effective and straightforward and would reduce healthcare costs and improve diagnosis response time and accuracy. [52]. In any case, the deployment of these algorithms is not exempt from controversies: hosting the AI models in a cloud service would entail uploading the images that might be subject to national and international regulations and constraints to ensure privacy [53].
